# Sprouty1 Regulates Reversible Quiescence of a Self-Renewing Adult Muscle Stem Cell Pool during Regeneration

**DOI:** 10.1016/j.stem.2009.12.015

**Published:** 2010-02-05

**Authors:** Kelly L. Shea, Wanyi Xiang, Vincent S. LaPorta, Jonathan D. Licht, Charles Keller, M. Albert Basson, Andrew S. Brack

**Affiliations:** 1 Massachusetts General Hospital, Center of Regenerative Medicine, Harvard University, Boston, MA, 02114, USA; 2 Department of Orthopedic Surgery, Massachusetts General Hospital, Boston, MA 02114, USA; 3 Division of Hematology/Oncology, Robert H. Lurie Comprehensive Cancer Center, Northwestern University Feinberg School of Medicine, Chicago, IL 60611, USA; 4 Department of Cellular & Structural Biology, The University of Texas Health Science Center, San Antonio, TX 78229, USA; 5 Department of Craniofacial Development, King's College London, Guy's Campus, London, SE1 9RT, UK

## Abstract

Satellite cells are a heterogeneous population of skeletal muscle specific stem cells capable of self-renewal and differentiation after transplantation. Whether quiescent satellite cells can self-renew and contribute to muscle fiber repair in their endogenous environment in normal regenerating muscle has remained unknown. The transcription factor Pax7 is expressed in satellite cells and is critical for establishing the adult satellite cell pool. Using a temporally-inducible genetic lineage tracing approach (*Pax7-CreER^tm^*; *R26R-lacZ)* to fate-map adult satellite cells, we show that in response to injury quiescent adult Pax7^+^ cells enter the cell cycle; a subpopulation return to quiescence to fully replenish the satellite cell compartment and the others contribute to *de novo* muscle fiber formation. We demonstrate that Sprouty1 (*Spry1*), an inhibitor of receptor tyrosine kinase signaling, is robustly expressed in quiescent Pax7^+^ satellite cells in uninjured adult muscle, down-regulated in proliferating myogenic cells in injured muscles, and re-induced as Pax7^+^ cells return to quiescence in regenerated muscles. We show through deletion of *Spry1* specifically in cycling adult Pax7^+^ satellite cells, that *Spry1* is required for the return to quiescence and homeostasis of the self-renewing Pax7^+^ satellite cell pool during repair. Satellite cells unable to return to quiescence succumb to apoptosis leading to a diminished self-renewing Pax7-derived satellite cell pool. Our results define a novel role for *Spry1* in adult stem cell biology and tissue repair.

## Introduction

Reversible quiescence is widely accepted to be a defining property of adult stem cells. The ability of adult stem cells to transition to a reversible quiescent state after providing a source of progeny is critical for homeostasis of tissue resident stem cells and presumably the maintenance of the tissue during numerous rounds of damage caused by various insults throughout life.

Adult skeletal muscle satellite cells (skeletal muscle stem cells) are located between the muscle fiber sarcolemma and the basal lamina surrounding the fiber and are major contributors to the maintenance and repair of postnatal skeletal muscle tissue. In uninjured muscle, satellite cells reside in a quiescent state and express the paired-box protein Pax7, a critical regulator of satellite cell survival and required for muscle tissue homeostasis (Seale et al., 2000; Oustanina et al., 2004; Kuang et al., 2006; Relaix et al., 2006). Transplantation studies have demonstrated that a sub-population of satellite cells are capable of both self-renewal and differentiation during muscle tissue repair (Montarras et al., 2005; Collins et al., 2005; Kuang et al., 2007; Sacco et al., 2008; Cerletti et al., 2008). However the mechanism by which a subset of satellite cells or their progeny by-passes cues to differentiate and instead return to quiescence to replenish the quiescent adult muscle stem cell pool, i.e., self-renew, during the regeneration process remains incompletely understood.

Receptor Tyrosine Kinase (RTK) signaling is critical for many processes during development and regeneration including myogenic fate decisions (Johnson et al., 1994; Poss et al., 2000; Lee et al., 2005; Abou-Khalil et al., 2009). RTK ligands such as FGF and HGF are potent activators of muscle satellite cells (Yablonka-Reuveni et al., 1999; Sheehan and Allen, 1999; Jones et al., 2001). The activity of these signaling pathways is tightly controlled by the action of feedback regulators. One downstream target and negative regulator of RTK signaling is Sprouty (*Spry*) (Hanafusa et al., 2002; Kim and Bar-Sagi, 2004; Mason et al., 2006). The mouse and human genomes contain four *Spry* genes (*Spry1-Spry4*). In *Drosophila*, *Spry* is required for cell fate determination in many settings (Reich et al., 1999; Kramer et al., 1999; Casci et al., 1999). Recent gain of function experiments demonstrated that *Spry2* promoted a shift in fate of myogenic cells, favoring a less differentiated Pax3/7-expressing population at the expense of a more differentiated myogenin-expressing population during embryonic myogenesis (Lagha et al., 2008). Microarray data suggested that quiescent adult satellite cells express high levels of *Spry1* with the other *Spry* family members present at much lower levels (Fukada et al., 2007). Importantly, *Spry1* expression was found to be down regulated in cycling myogenic progenitors, raising the possibility that *Spry1* normally inhibits the RTK signals required for the proliferation of these cells.

We used genetic lineage tracing to determine whether the quiescent adult Pax7 cell is capable of replenishing the renewed satellite cell pool and contributing to myofiber regeneration. In addition, we sought to determine whether *Spry1* is required for homeostasis of the endogenous adult Pax7 satellite cell pool in their native environment during muscle regeneration. We show that the endogenous population of adult Pax7 cells is capable of replenishing the satellite cell pool and expands to provide a source of differentiated progeny contributing to muscle fiber repair. Investigation into the role of *Spry1* revealed that appropriate regulation of reversible quiescence is essential for self-renewal of the adult Pax7-expressing satellite cell pool during muscle regeneration.

## Results

### Endogenous adult Pax7 cells function as muscle stem cells within their native environment during regeneration

**Figure 1:**
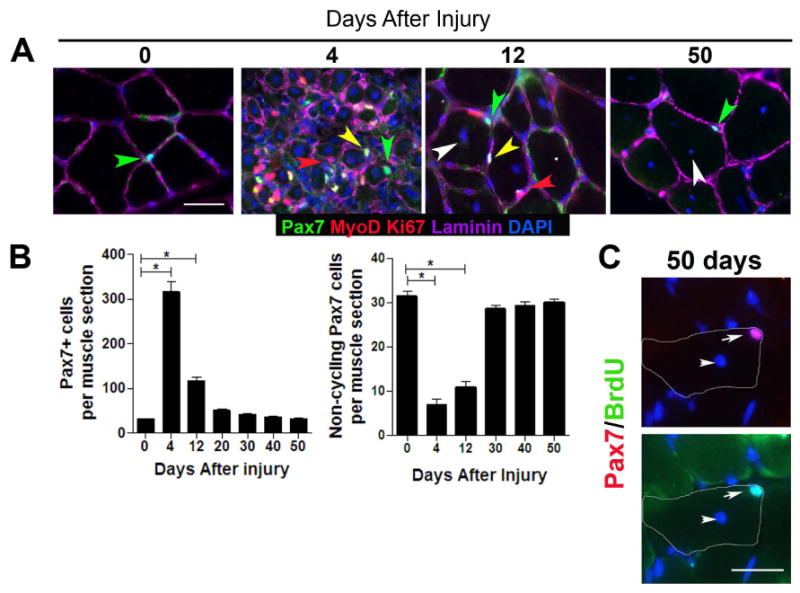
The pool of quiescent Pax7 satellite cells returns to homeostasis during adult muscle regeneration (A) Transverse sections of uninjured (0 days after injury) and regenerating (4, 12, and 50 days after injury) TA muscle contain quiescent (MyoD^-^, Ki67^-^) (green arrowhead) and cycling (MyoD^+^, Ki67^+^; yellow arrowhead) Pax7^+^ cells in sublaminar location (laminin^+^; magenta). Differentiating myogenic cells (red arrowhead) could be observed in regenerating muscle underneath the basal lamina. (B) Total number of Pax7^+^ cells (left) and quiescent (Ki67^-^; MyoD^-^) Pax7^+^ cells (right) per muscle section from 4-6 mice per time point (mean ± sem; *p*<0.05). (C) BrdU^+^/Pax7^+^ cells in transverse sections from fully regenerated muscle from BrdU-treated mice. Top panel: Pax7 (red) and DAPI (blue) staining, Bottom panel: BrdU (green) and DAPI staining. Regenerated muscle fibers are distinguished with DAPI^+^ central nuclei (white arrowhead). The contour of muscle fiber is denoted by white line. Scale bar; 40 μm (A), 80 μm (C).

Pax7 is a marker of adult satellite cells and is required for the formation of the adult satellite cell pool (Seale et al., 2000; Oustanina et al., 2004). The ability of adult skeletal muscle to undergo numerous rounds of repair after injury suggests that self-renewal occurs and homeostasis of the adult muscle satellite cell pool is restored or otherwise maintained during muscle regeneration after injury. To test this, we quantified the number of Pax7^+^ cells within adult skeletal muscle during regeneration. *Tibialis Anterior/Extensor Digitorus Longus* (TA/EDL) muscle was injected with 50μl of 1.2% barium chloride and left to regenerate from 4-50 days (Figure 1A, S1). Immunohistochemical analysis revealed a transient expansion of the number of cycling (MyoD^+^/Ki67^+^) (Zammit et al., 2004; Sacco et al., 2008) Pax7^+^ cells and a diminished quiescent (MyoD^-^/Ki67^-^) Pax7^+^ pool between 4-12 days after injury compared to uninjured muscle (Figure 1B). Between 30-50 days after injury, the quiescent pool of Pax7^+^ cells, which represented 98% of the total Pax7 pool, returned to homeostatic levels that were observed in uninjured muscle. To determine whether Pax7^+^ cells that re-occupy the satellite cell position in regenerated fibers had previously cycled, BrdU was administered during the first 7 days of regeneration and analyzed in Pax7^+^ cells. Muscle sections from fully regenerated muscle showed that 98±0.4% of quiescent Pax7^+^ cells (1493/1500, n=4 mice) had incorporated and retained BrdU and therefore had previously cycled (BrdU^+^) during regeneration (Figure 1C). In contrast, Pax7^+^ cells in uninjured muscle did not have detectable BrdU (data not shown). This demonstrates that under the injury paradigm used in the present study, the quiescent Pax7^+^ satellite cell pool homogeneously enters the cell cycle during repair, and that both the quiescent state and the size of the Pax7^+^ satellite cell pool have been fully restored to that of uninjured muscle upon completion of muscle repair. This suggests that the quiescent Pax7^+^ satellite cell pool is reversibly quiescent and is under homeostatic control during muscle regeneration. However, this type of analysis cannot exclude the possibility that Pax7^+^ satellite cells in repaired muscle are derived from another source that subsequently acquired a Pax7 phenotype.

**Figure 2:**
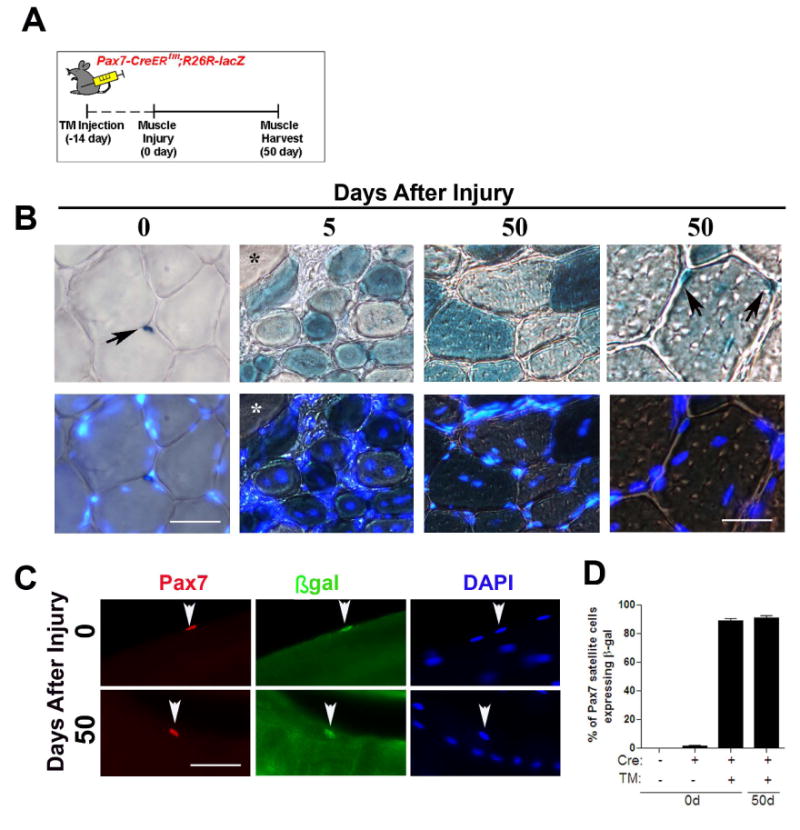
Pax7-derived satellite cells are capable of self-renewal and differentiation (A) The cartoon depicts the tamoxifen (TM) injection scheme for lineage tracing muscle satellite cells. Pax7^+^ satellite cells in the *Pax7-CreER^tm^;R26R-lacZ* mouse were permanently labeled by IP administration of TM 14 days prior to muscle injury. (B) Transverse sections were collected from uninjured and regenerating muscle and stained with X-gal. Five days after injury non centrally-nucleated fibers in regenerating muscle had no detectable X-gal reactivity (*). After 50 days of regeneration, X-gal^+^ mononucleated cells were observed in the satellite cell position of regenerated muscle fibers (black arrows). (C) Single fibers from uninjured and regenerated *Pax7-CreER^tm^;R26R-lacZ* muscle stained with Pax7 (red), β-gal (green) and DAPI (blue). Regenerated muscle fibers are characterized by DAPI^+^ ‘central-myonuclear chains’. (D) The percentage of β-gal^+^/Pax7^+^ satellite cells expressing on single fibers isolated from uninjured and regenerated muscle fibers in the presence (+) or absence (-) of Cre or TM. Data is presented as mean ±sem. n=4-6 mice. (*p*<0.05). Scale bars in (B) are 60 μm (left) and 30 μm (right) and 40 μm in (C).

To investigate directly whether quiescent adult Pax7^+^ satellite cells have stem cell properties that enable self-renewal and therefore both return to quiescence as well as contribute to muscle fiber formation (differentiation) in their endogenous environment, we used mice with an inducible *Pax7-CreER^tm^* (Nishijo et al., 2009) and a *R26R-lacZ* reporter (Soriano, 1999) to genetically lineage trace adult Pax7^+^ cells. Tamoxifen (TM) was administered to induce gene recombination and permanently express β-gal in Pax7^+^ cells and their progeny. TA/EDL muscles from one leg were injured and left to regenerate and the contra-lateral leg remained uninjured (Figure 2A). In transverse sections from the uninjured muscle, X-gal reactivity was observed in mononucleated cells located in the satellite cell position. In the regenerating muscle, X-gal reactivity was observed in small, *de novo* muscle fibers but not in adjacent uninjured fibers. This suggests that adult Pax7-derived satellite cells are able to contribute to muscle differentiation *in vivo*. Fifty days after injury, X-gal^+^ mononucleated cells were readily detected in the satellite cell position of regenerated (centrally-nucleated) muscle fibers (Figure 2B). As confirmation that β-gal expression was a readout of Pax7-derived satellite cells, immunohistochemical analyses from isolated single muscle fibers from TM treated *Pax7-CreER^tm^*;*R26R-lacZ* mice showed that β-gal expression was observed in ∼84% and ∼87% of Pax7^+^ satellite cells from uninjured and regenerated muscle, respectively (Figure 2C, 2D). Together, this data suggests that adult Pax7^+^ cells are capable of self-renewal and differentiation in their endogenous environment, and are sufficient to replenish the satellite cell pool in the regenerating muscle.

### Sprouty1 expression is restricted to non-cycling Pax7 satellite cells

**Figure 3:**
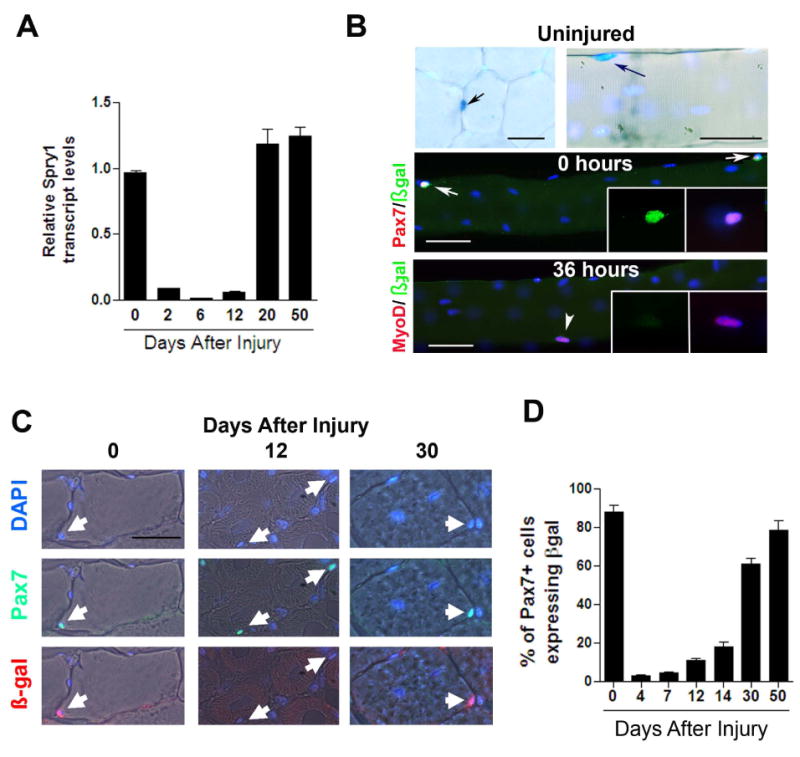
Sprouty1 is a marker of quiescent Pax7^+^ satellite cells (A) Relative *Spry1* transcript levels assessed by real-time qRT-PCR from FACS-purified myogenic cells (See Figure S3 for FACS sorting and Ct values) isolated from uninjured and regenerating muscle. (B) Transverse sections and single muscle fibers from uninjured muscles of *Spry1^lacZ/^*^+^ mice were stained with X-gal (upper panels). X-gal^+^ mononucleated cells (blue; black arrows) were observed in the satellite cell position in muscle sections (left) and single fibers (right). Single fibers from *Spry1^lacZ/^*^+^ mice were stained with anti-β-gal (green) and Pax7 (red) (middle row; white arrows) or β-gal (green) and MyoD (red) (bottom row; white arrowhead) after 0 hours or 36 hours in culture. Inset show β-gal^+^/Pax7^+^ cell (middle row) and β-gal^-/^ MyoD^+^/ cell (lower row). (C) Muscles from *Spry1^lacZ^*^+^*^/-^* were injured and left to regenerate from 4 to 50 days or remained uninjured. Muscle sections stained with anti-Pax7 and β-gal is shown from uninjured and regenerating muscle. Arrows show Pax7^+^/β-gal^+^ cells (at 0 and 30 days after injury) and Pax7^+^/β-gal^-^ cells (at 12 days after injury). (D) Quantitation of (C), showing the percentage of β-gal^+^/Pax7^+^ cells in muscle sections. Data were presented as mean ± sem. n=4-6 mice. Scale bar in (B) is 80 μm and (C) is 40 μm.

As RTK/MAPK signaling has been implicated in the process of quiescence and self-renewal (Jones et al., 2005; Abou-Khalil et al., 2009), we characterized the expression dynamics of a candidate gene, *Spry1* (Sprouty1), which encodes a RTK/MAPK regulator (Kim and Bar-Sagi, 2004) and is abundantly expressed in muscle satellite cells (Fukada et al., 2007). Myogenic cells were obtained by fluorescent activated cell sorting (FACS) from uninjured and regenerating adult muscle (Figure 3A, S3). *Spry1* transcript was abundantly expressed in myogenic cells isolated from uninjured (0 days) and regenerated muscle (20 and 50 days), when most myogenic cells are non-cycling (Figure 3A, 1A, S3B). *Spry1* levels were reduced in myogenic cells isolated from muscle tissue at 2, 6 and 12 days after injury, when myogenic cells are proliferating or differentiating (Figure 3A, 1A, S3B). Therefore, *Sprouty1* expression in myogenic cells is temporally correlated with a quiescent state and is down regulated during the proliferative phase of muscle regeneration. To characterize *Spry1* expression in muscle on a cell-by-cell basis, we analyzed mice carrying one copy of a *Spry1lacZ* knockin allele (*Spry1^lacZ/^*^+^) (Thum et al., 2008) as a readout of *Spry1* expression. In muscle sections from uninjured adult mice, X-gal^+^ cells were primarily detected in the characteristic satellite cell position (Figure 3B). Single muscle fibers isolated from *Spry1^lacZ/^*^+^ muscle were fixed immediately (0 hours) or cultured for 36 hours to activate satellite cells (Zammit et al., 2004). β-gal was expressed in 92% of Pax7^+^ cells (n=184/197) at 0 hours and in only 8% (n=16/202) of activated (MyoD^+^) satellite cells after 36 hours in culture. We next analyzed β-gal expression in Pax7^+^ satellite cells of *Spry1^lacZ/^*^+^ muscle during muscle regeneration *in vivo* (Figure 3C). *Spry1* as determined by β-gal expression was robustly expressed in Pax7^+^ cells when they are primarily quiescent in uninjured muscle or returning to quiescence during muscle regeneration (Figure 3D, 1B, S3B). These results confirmed that *Sprouty1* expression was restricted to Pax7^+^ cells when they are in a quiescent state.

### Sprouty1 is required for self-renewal of the quiescent Pax7-derived satellite cell pool during regeneration

**Figure 4:**
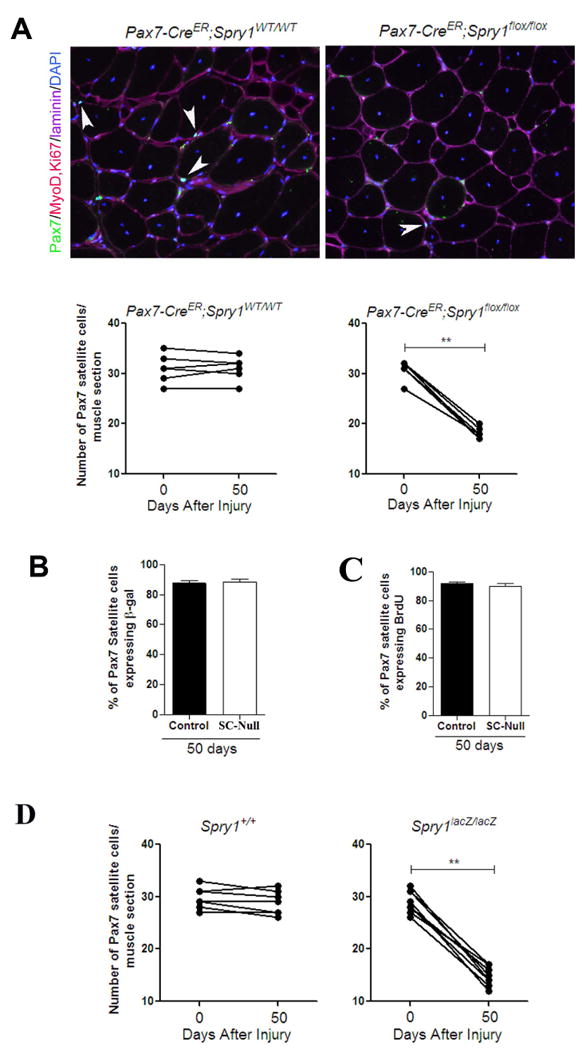
Sprouty1 is required for restoring the muscle stem cell pool during regeneration (A) Mice were injected with TM to permanently activate Cre in *Pax7-CreER^tm^;Spry1^flox/flox^; R26R-lacZ* (SC-Null) and *Pax7-CreER^tm^;Spry1^WT/WT^*;*R26R-lacZ* (Control) adult mice. 14 days after TM treatment TA/EDL muscles from one leg were injured and left to regenerate for 50 days. Muscle sections stained with anti-Pax7, MyoD, Ki67, laminin and DAPI (Upper panels is shown from Control and SC-Null regenerated muscle; white arrow heads denote sub-laminar Pax7 cells). Graphs (lower panels) present the average number of Pax7^+^ cells in sub-laminar position. The bar between each point defines each animal. (*p*<0.01). (B) Single muscle fibers from (A) were stained with anti-Pax7 and β-gal to quantify the percentage of Pax7^+^ cells expressing β-gal driven from the *Pax7-Cre* reporter. (C) BrdU was administered in (A) during first 10 days after muscle injury. Muscle sections were stained with anti-BrdU, Pax7 and laminin. Histogram shows the percentage of BrdU^+^/Pax7^+^ cells satellite cells after 50 days of regeneration. (D) Muscle sections from *Spry1*^+^*^/^*^+^ and *Spry1^lacZ/lacZ^* were stained with anti-Pax7, MyoD, Ki67 and laminin (see Figure 4A). Graphs show the average number of Pax7^+^ cells in sub-laminar position in uninjured and regenerated muscle sections of individual mice. The bar between each point defines each animal. Scale bar; 40 μm.

Having established that *Cre* expression was induced in resting adult satellite cells and could be used to follow cell fate in combination with a reporter gene, we employed this system to determine the molecular regulators of satellite cell self-renewal and homeostasis. To directly test the role of Sprouty1 in Pax7^+^ satellite cells during muscle regeneration. We produced *Pax7-CreER^tm^*;*Spry1^flox/flox^*;*R26R-lacZ* (termed, Satellite Cell-specific Null (SC-Null)) mice to permanently disrupt *Spry1* function in adult Pax7^+^ satellite cells upon the administration of TM. *Pax7-CreER^tm^*;*Spry1^WT/WT^*;*R26R-lacZ* mice treated with TM were used as controls. A high degree of recombination was evident in FACS-purified myogenic cells from SC-Null mice based on expression of the *R26R-lacZ* reporter (89% X-gal^+^/myogenic cells) and disruption of *Spry1* at both the gene and transcription level (Figure S4). To determine whether *Spry1* was required for the return to quiescence of the Pax7 satellite cell pool after muscle injury, the total number of Pax7^+^ cells and their cycling status, based on MyoD and Ki67 immunohistochemistry, were determined in TA/EDL muscles from Control and SC-Null mice that were injured and allowed to regenerate for 50 days. Contra-lateral muscles that remained uninjured served as internal controls. In Control animals, the presence of Cre or activation of Cre by TM did not change the number of Pax7^+^ cells in the regenerated muscle compared to the uninjured contra-lateral muscles (Figure 4A), indicating that muscle stem cell homeostasis was restored after muscle injury as shown previously (Figure 1A). However, in SC-Null muscle, there was a 40% decline in the number of Pax7^+^ satellite cells after 50 days of regeneration compared to the uninjured contra-lateral muscle and the regenerated Control muscles (Figure 4A), suggesting *Spry1* is essential for homeostasis of the replenished satellite cell pool. To test whether the remaining Pax7^+^ cells in SC-Null regenerated muscle were not targeted by Cre or had arisen from a non-Pax7 origin, we quantified β-gal expression that represented Pax7-Cre activity prior to muscle injury. In regenerated Control and SC-Null muscles, 87% and 88% of Pax7^+^ cells were β-gal^+^ respectively, confirming the majority of the replenished Pax7^+^ satellite cell pool was derived from cells of a Pax7 origin (Figure 4B). In uninjured muscle, *Spry1* disruption does not lead to a loss of Pax7^+^ satellite cells, therefore we tested whether the remaining Pax7^+^ cells in the regenerated SC-Null muscle responded to the injury stimulus by entering the cell cycle. In both Control and SC-Null muscle, ∼90% of Pax7^+^ cells that replenished the satellite cell niche of the regenerated muscle had entered the cell cycle during regeneration as indicated by BrdU incorporation (Figure 4C) and had subsequently returned to quiescence (97±4% and 98±1% of Pax7^+^ cells were MyoD^-^/Ki67^-^ in Control and SC-Null muscle, respectively). To confirm that the complete loss of *Spry1* would result in a reduced pool of Pax7^+^ satellite cells after injury, we injured adult muscles from *Spry1^lacZ/lacZ^* mice that lack Spry1 protein (Figure S5) and their control littermates, *Spry1*^+^*^/^*^+^. Fifty days after injury the number of Pax7^+^ satellite cells in *Spry1^lacZ/lacZ^* was reduced by 50% compared to *Spry1*^+^*^/^*^+^ Controls (Figure 4D). These results indicate that homeostasis of the quiescent satellite pool in the absence of *Spry1* is not fully restored after injury and that *Spry1* is essential for self-renewal of approximately half of the Pax7-derived muscle stem cell pool during muscle regeneration.

### Sprouty1 regulates a balance between reversible quiescence and apoptosis

**Figure 5:**
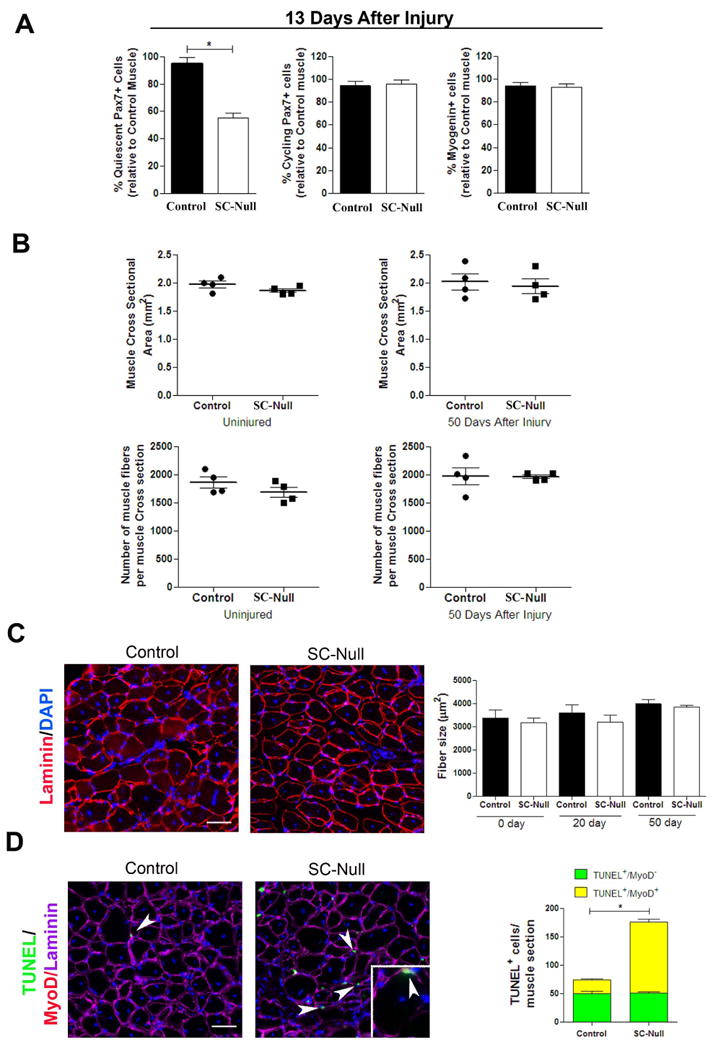
Disruption of *Spry1* in Pax7 cells affects apoptosis but not differentiation of myogenic cells during regeneration (A) Quantitative analysis of regenerating muscle (13 days after injury) from TM-treated *Pax7-CreER^tm^;Spry1^WT/WT^* (Control) and *Pax7-Cre^ER^;Spry1^flox/flox^* (SC-Null) mice. Graph shows the average number of quiescent (MyoD^-^, Ki67^-^) (left panel), cycling (MyoD^+^, Ki67^+^) (middle panel) Pax7^+^ cells and differentiated myogenic cells (Myogenin^+^) (right panel) in serial sections of TA muscle. Cell counts are expressed relative to Control muscle. Data were averaged (n=3 mice per group) and expressed as mean ±sem. (*p*<0.05). (B) Quantitative analysis of uninjured and regenerated muscle from TM-treated Control and SC-Null mice. Graph shows the average muscle fiber cross-sectional area (top panels) and the total number of muscle fibers (bottom panels) of uninjured and regenerated muscle in serial sections from the mid-belly of TA muscle. (C) TM treated Control (*Pax7-CreER^tm^;Spry1^WT/WT^*) and SC-Null (*Pax7-CreER^tm^;Spry1^flox/flox^*) muscle at 50 days after injury stained with laminin and DAPI (left panels). Histogram shows the average muscle fiber size in uninjured and regenerated muscle (n=4 mice per group) and expressed as mean ±sem. (D) Control and SC-Null muscles described in (C), were stained for TUNEL (green), MyoD (red), laminin (magenta) and DAPI (left panels). TUNEL^+^ cells are located sub- and peri-laminar position (white arrowheads). Inset: TUNEL^+^/MyoD^+^ nucleus (yellow) located underneath the basal lamina of a regenerating muscle fiber (white arrowhead). Histogram (right) shows the number of TUNEL^+^, MyoD^+^ (yellow) and MyoD^-^ (green) cells per muscle section. Data were averaged (4 mice per group) and expressed as mean ±sem. (*p*<0.05). Scale bar; 80 μm.

A failure of satellite cells to return to quiescence in the absence of *Spry1* may be due to either an inability to fully expand the pool of cycling Pax7 cells or altering the fate of these cells during regeneration from a self-renewing fate to differentiation or apoptosis. To test this, TA/EDL muscle from TM-treated Control and SC-Null mice were injected and left to regenerate for 13 days. Muscle sections were analyzed for the number of Pax7^+^ cells and differentiated myogenic cells. In SC-Null muscle the percentage of quiescent Pax7^+^ cells was reduced by 40%, whereas the percentage of cycling Pax7^+^ cells and differentiated (Myogenin^+^) cells was not altered compared to Control muscles (Figure 5A, S6). We next analyzed the number and size of muscle fibers as well as the number of apoptotic myogenic cells in uninjured and regenerating muscle from Control and SC-Null mice. The total cross-sectional area of muscle, the total number of muscle fibers (Figure 5B), and the average muscle fiber size were not different between SC-Null and Control muscles at either 20 or 50 days after injury compared to regenerating Control muscle (Figure 5C) suggesting that Pax7^+^ cells with disrupted *Spry1* function were not favoring differentiation at the expense of stem cell homeostasis and that *Spry1* was not required for myogenic differentiation *in vivo*. In contrast, there was an increased number of apoptotic myogenic cells (TUNEL^+^/MyoD^+^) in SC-Null compared to Control regenerating muscle analyzed 20 days after injury (Figure 5D). Therefore, Sprouty1 is essential for self-renewal and homeostasis of the quiescent satellite cell pool but dispensable for muscle fiber differentiation during regeneration.

### Sprouty1 is required for myogenic progenitors to return to quiescence

**Figure 6:**
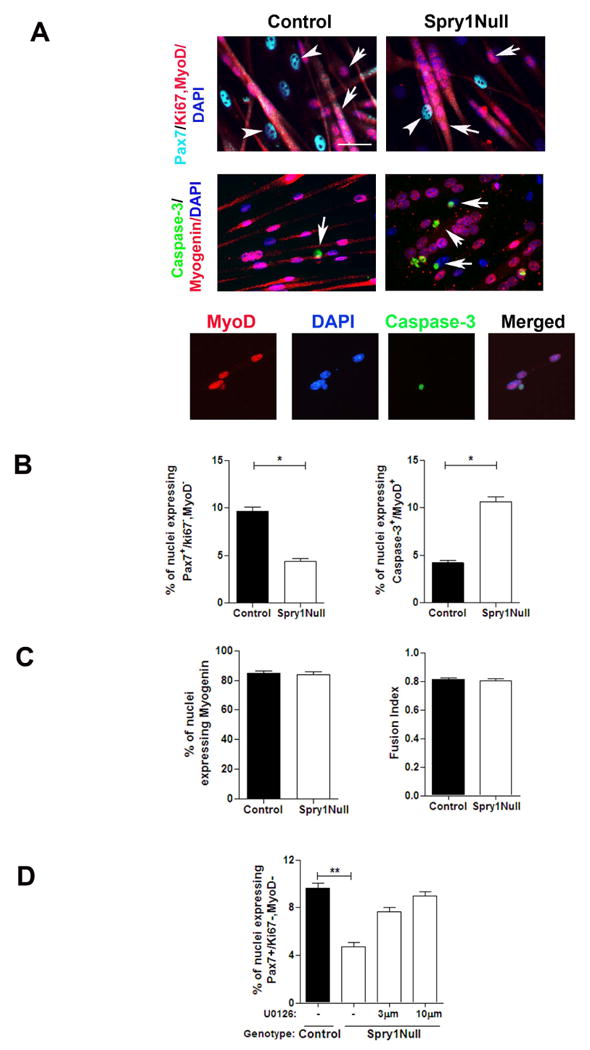
Sprouty1 regulates reversible quiescence of a subset of satellite cell progenitors (A) Primary myoblasts from *Spry1^WT/WT^*;*R26R-lacZ* and *Spry1^flox/flox^:R26R-lacZ* mice were incubated with Cre adenovirus (Control and Spry1Null respectively) and switched to low mitogen media. Cultures were stained with markers to determine quiescence (top panel; Pax7^+^, MyoD^-^, Ki67^-^), differentiation (middle panel; Myogenin^+^) and apoptosis (middle panel; activated Caspase-3). White arrowheads show quiescent cells; white arrows show differentiated (top panels) and apoptotic (middle panels) cells. Lower panel shows activated Caspase-3 staining in mononucleated MyoD^+^ cells. (B) Histograms show that the percentage of quiescent Pax7^+^ cells (left), percentage of apoptotic cells (right) and (C) percentage of differentiated myogenic cells (left) and ability to fuse (right). (D) MEK inhibitor, U0126 (3 and 10 μM) or diluent alone (DMSO (-)) was added to *Spry1^flox/flox^* (Spry1Null) and *Spry1^WT/WT^* (Control) reserve cell cultures for 2 days. Histogram shows the percentage of quiescent (Pax7^+^, MyoD^-^, Ki67^-^) cells. Scale bar; 40 μm.

The time-course of *Spry1* gene expression during regeneration indicated that *Spry1* is rapidly down regulated in cycling satellite cells after injury and is induced again 12 days after injury coinciding with the re-emergence of the quiescent phenotype of Pax7^+^ satellite cells (Figure 1, 3). We therefore asked whether *Spry1* was required for returning a subset of cycling myogenic cells to quiescence. Using single muscle fiber suspension cultures, we analyzed the number of muscle fiber-resident Pax7^+^ cells that returned to quiescence (re-quiescence) after cycling (i.e., self-renew) (Zammit et al., 2004). The total number of Pax7^+^ cells per single muscle fiber was not different between TM treated SC-Null cultures and Control muscle fiber cultures (Figure S7). In contrast, the number of quiescent Pax7^+^ cells per fiber was reduced by 50% in SC-Null compared to Control cultures. These data provide evidence that *Spry1* is required for reversible quiescence of a sub-population of Pax7^+^ cells. We next examined the role of *Spry1* in regulating re-quiescence of satellite cell progenitors using an *in vitro* ‘reserve’ cell preparation (Yoshida et al., 1998; Perez-Ruiz et al., 2008) (Figure 6). Low passage primary myoblasts from *Spry1^flox/flox^* and *Spry1^WT/WT^* muscle were infected with Cre or Control adenovirus, left to recover for 24 hours and switched to low serum conditions for 3.5 days. This method reliably achieved 97% infection/recombination efficiency (Figure S8). Based on immunocytochemistry, myogenic cells in low serum cultures were characterized as quiescent (Pax7^+^, MyoD^-^ and Ki67^-^), apoptotic (Activated-Caspase-3^+^) and differentiated (Myogenin^+^) (Figure 6A). After 3.5 days in culture, the percentage of quiescent myogenic cells decreased and the percentage of apoptotic cells increased in Cre-infected *Spry1^flox/flox^* cells (Spry1Null) compared to Cre-infected *Spry1^WT/WT^* cells (Control, Figure 6B) and cells treated with Control virus (data not shown). Neither the percentage of cells expressing the differentiation marker, Myogenin, nor their ability to fuse was altered by disruption of *Spry1* (Figure 6C). This observation supports our hypothesis that *Spry1* is essential for a subset of myogenic progenitors to return to a quiescent state, whilst *Spry1* appears redundant for myogenic differentiation. Furthermore, cells expressing the activated form of Caspase-3 were almost exclusively restricted to the MyoD^+^/Myogenin^-^ population of progenitor cells, suggesting that a cell fate decision between re-quiescence and apoptosis occurs in myogenic progenitors prior to committing to terminal differentiation.

Consistent with Spry's role as an inhibitor of ERK signaling (Kim and Bar-Sagi, 2004), we observed that ERK signaling was up regulated in myogenic cells lacking *Spry1* (Figure S5). We tested whether the inability of cycling myogenic cells to return to quiescence in the absence of *Spry1* as shown in Figure 6B, was due to elevated ERK signaling. Cre adenovirus-treated *Spry1^flox/flox^* (Spry1Null) and *Spry1^WT/WT^* (Control) cultures were incubated in low serum conditions to generate ‘reserve’ cells, in the presence or absence of the MEK inhibitor, U0126. After 3 days in culture, cells were fixed and analyzed for their quiescence immunophenotype (Figure 6D). The percentage of quiescent cells increased in Spry1Null cells in the presence of the MEK inhibitor. Moreover, the percentage of quiescent cells in Spry1Null cultures treated with U0126 was indistinguishable from Control virus treated *Spry1^WT/WT^* cells in the absence of U0126. Together, this suggests that ERK signaling regulates cell fate decisions involving reversible quiescence and that Sprouty1 functions primarily through an ERK-dependent signaling cascade to regulate myogenic progenitor re-quiescence.

### Sprouty1 regulates homeostasis of the self-renewing satellite cell pool through a subset of cycling Pax7-derived satellite cells

**Figure 7:**
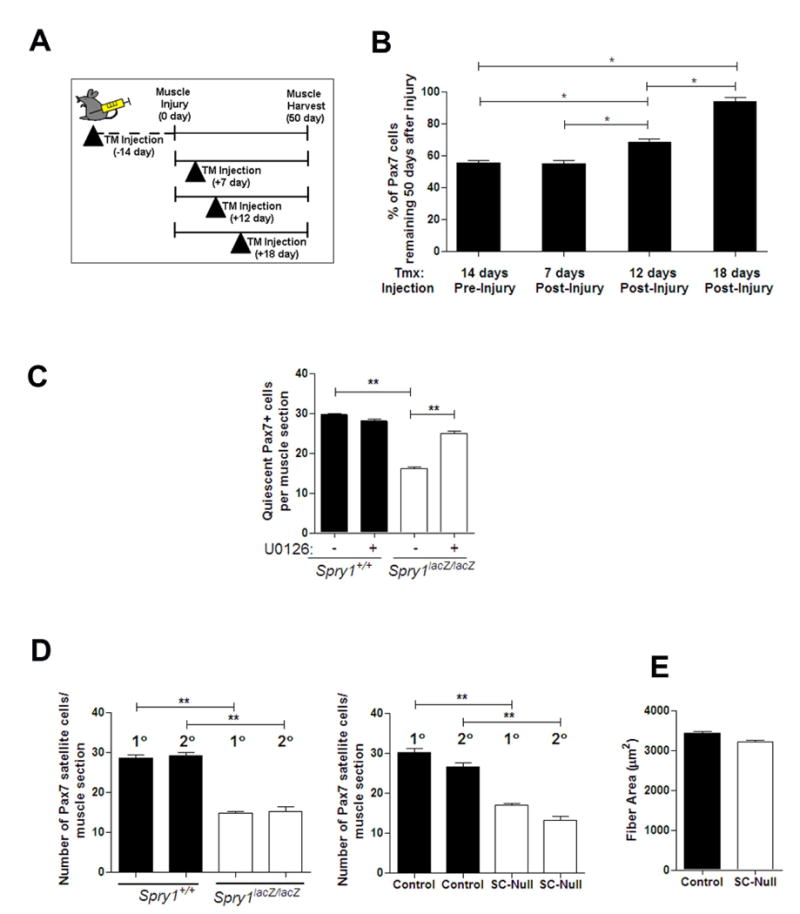
Return to quiescence and self-renewal is temporally instructed in a subpopulation of cycling Pax7 satellite cells (A) Cartoon depicting the TM strategy to induce Cre activation in Control (*Pax7-CreER^tm^;Spry1^WT/WT^*) and SC-Null (*Pax7-CreER^tm^;Spry1^flox/flox^*) mice 14 days prior to injury) or at distinct phases of muscle regeneration. Muscles were analyzed 50 days after muscle injury. (B) Muscle sections from experiments depicted in (A) were stained with anti-Pax7, MyoD, Ki67, laminin and DAPI. Histogram shows the average number of Pax7^+^ cells per muscle section in regenerated SC-Null muscle expressed relative to the number of Pax7^+^ cells in the uninjured contra-lateral muscle. Data were averaged over a minimum of 4 mice per group and expressed as mean ± sem. (C) Muscles from *Spry1*^+^*^/^*^+^ and *Spry1^lacZ/lacZ^* adult mice were injured and left to regenerate for 13 days. Mice were treated with U0126 or vehicle (DMSO) via IP injection 10 and 11 days after injury. Histograms show the number of quiescent Pax7^+^ satellite cells located underneath the basal lamina of muscle sections. (D) Mice were subjected to two rounds of injury (1° and 2°) and 50 days of repair. Fifty days after the second injury muscle was analyzed for the number of Pax7^+^ cells in satellite cell position in *Spry1*^+^*^/^*^+^ and *Spry1^lacZ/lacZ^* (left panel) and TM-treated SC-Null and littermate controls (right panel). (**,*p*<0.01). (E) The average muscle fiber size from Control and SC-Null muscles from (D).

Our data so far suggest that *Spry1* function is required to maintain the quiescent stem cell pool during muscle repair. We next wanted to test directly whether *Spry1* was required for cycling Pax7 satellite cells to re-establish the quiescent Pax7^+^ satellite cell pool back to homeostasis during regeneration. We first determined whether satellite cells that re-occupy the satellite cell position in regenerated muscle are from a previously cycling population. When BrdU was administrated from 0-7 days after injury, there were ∼98% of Pax7^+^ cells retaining BrdU label in fully regenerated TM-treated Control and SC-Null muscle (490 Pax7^+^/500 BrdU^+^ and 583 Pax7^+^/600 BrdU^+^ respectively, n=4 individual mice per genotype), suggesting nearly all Pax7^+^ cells in regenerated muscle are derived from Pax7^+^ satellite cells that have divided during repair. To identify exactly when *Spry1* was required for satellite cell re-quiescence during muscle regeneration *in vivo*, we injected TM into SC-Null and Control mice at distinct times during muscle regeneration (Figure 7A) and quantified the number of Pax7^+^ satellite cells from serial sections of uninjured and regenerated muscle (Figure 7B). TM was injected between 16-14 days prior to injury to disrupt *Spry1* function in quiescent Pax7^+^ cells and their progeny or after injury to target Pax7^+^ cells in injured muscle that had entered the cell cycle. In regenerated muscle from TM-treated Control mice, the average number of Pax7^+^ cells per muscle section was not significantly different from the contra-lateral uninjured muscle. TM injected into SC-Null mice 14 days prior to injury resulted in a 40% loss in the size of the Pax7^+^ satellite cell pool in fully regenerated muscle. Remarkably, a 40% loss in the size of the self-renewed Pax7^+^ pool was also observed when TM was injected into SC-Null mice 7-10 days after muscle injury, thus not affecting *Spry1* function in quiescent Pax7^+^ cells prior to injury (Figure 7B). The result is consistent with a subset of Pax7-derived progenitors providing a source of satellite cells that require *Spry1* expression for their return to quiescence and homeostasis of the self-renewing Pax7 pool. To test whether the influence of *Spry1* on restoration of the muscle stem cell pool was temporally restricted during regeneration, we injected TM later during muscle regeneration (10-12 or 18-20 days after injury). The effect of *Spry1* disruption at later time point was more subtle than injections at earlier time points, reducing the number of Pax7^+^ satellite cells that replenished the regenerated muscle by ∼30% and ∼5% respectively, compared to uninjured contra-lateral muscle. The results are consistent with the critical role of Sprouty1 in conveying temporally coordinated signals within the regenerating muscle in a subset of Pax7^+^ satellite cell descendents that possess self-renewal properties and are essential for re-establishing homeostasis of the Pax7^+^ satellite cell pool during repair.

We next asked whether inhibition of ERK signaling could prevent the diminution of the quiescent Pax7^+^ satellite cell pool in the absence of *Spry1* during muscle regeneration. TA/EDL muscles from *Spry1*^+^*^/^*^+^ and *Spry1^lacZ/lacZ^* were injured and allowed to recover for 13 days. Mice were administered U0126 or vehicle via IP injection at 11 and 12 days after injury. The number of quiescent Pax7^+^ cells increased in U0126-treated *Spry1^lacZ/lacZ^* muscle in comparison to that observed in Control muscle (Figure 7C). Therefore, Spry1 signals through the ERK cascade to regulate reversible quiescence of the Pax7 satellite cell pool during muscle regeneration.

The results so far are consistent with a specific sub-population of quiescent Pax7-derived satellite cells requiring *Spry1* for their self-renewal. We reasoned that the self-renewing *Spry1*-“independent” Pax7^+^ satellite cell pool would self-renew as effectively as Pax7-derived satellite cells from Control muscles. To test this hypothesis we asked whether upon a second round of injuries the Pax7^+^ pool in *Spry1*-deficient satellite cells was diminished. At fifty days after a primary injury, TA/EDL muscle from *Spry1^lacZ/lacZ^*, SC-Null and their respective Control muscles were exposed to a secondary injury and allowed to recover for a further 50 days. The number of Pax7^+^ satellite cells (Figure 7D) and average muscle fiber size (Figure 7E) was not altered after a secondary injury compared to the primary injury in *Spry1* loss-of-function mutants, similar to their controls. Together, these results strongly suggest that Sprouty1 is essential for the return to quiescence of a distinct sub-population of self-renewing Pax7^+^ muscle stem cells during muscle regeneration.

## Discussion

We undertook a genetic lineage tracing analysis in combination with a temporally inducible, cell specific gene deletion approach to identify the endogenous pool of adult muscle stem cells within their native environment, as well as to determine the molecular regulators of their self-renewal capacity during muscle regeneration. The results of the current study demonstrate that adult Pax7-expressing satellite cells can contribute to both *de novo* myofiber formation and restoration of the satellite cell pool back to homeostatic levels after injury. Our studies illustrate a novel role of the RTK inhibitor, Sprouty1 (*Spry1*) in adult stem cell function and tissue homeostasis. We have identified that disruption of *Spry1* in adult Pax7^+^ cells prevents their return to quiescence and results in a failure to replenish the satellite cell pool to homeostatic levels after muscle injury. Spry1 function was limited to a sub-population of Pax7-derived cells able to control the size of the self-renewing muscle stem cell pool but not their differentiation potential. Together, these results demonstrate that *Spry1* is an essential regulator of quiescence and homeostasis of the adult muscle stem cell pool during muscle regeneration.

Genetic lineage tracing approaches offer an alternative method to transplantation assays to track endogenous stem cell populations and their functional potential within their native environment. Lineage tracing experiments have shown that nearly all (> 90%) adult quiescent satellite cells have expressed transcription factors, Pax3, Myf5, MyoD and Pax7 at some point during their developmental history (Schienda et al., 2006; Kuang et al., 2007; Kanisicak et al., 2009; Lepper et al., 2009; Brack et al., 2009). By using an inducible *Pax7 Cre* allele, it was recently shown that adult Pax7^+^ satellite cells are capable of contributing to muscle fiber repair after multiple rounds of injury, thus demonstrating their stem cell potential (Lepper et al., 2009). Using a different inducible *Pax7-Cre* allele (Nishijo et al., 2009), we demonstrate that upon barium chloride-induced muscle injury Pax7-derived cells contribute to myofiber differentiation and fully restore the renewed satellite cell pool back to homoeostasis during repair.

We demonstrate that Spry1, an inhibitor of growth factor signaling, is robustly and preferentially expressed in Pax7^+^ cells in their quiescent state in uninjured and regenerating muscle *in vivo*, suggesting that *Spry1* is a marker of Pax7^+^ satellite cells in their quiescent state (Fukada et al., 2007). Low levels of *Spry1* expression could be observed in subsets of nascent myotubes *in vivo* and *in vitro* (data not shown) as shown previously (Laziz et al., 2007). This likely reflects that *Spry* genes are activated in the presence of growth factors within the extrinsic milieu and that differentiated myotubes can respond to growth factor signals (Perez-Ruiz et al., 2007). That *Spry1* expression is elevated and sustained in quiescent Pax7^+^ cells compared to either cycling Pax7^+^ cells or differentiating myogenic cells suggests specificity in Spry1 signaling components within myogenic cells of different fates.

The ability of a cell to undergo reversible quiescence is critical for maintenance of the stem cell pool (Sang et al., 2008). Depletion of the stem cell pool is often observed in conditions of maintained proliferation (Orford and Scadden, 2008) and may explain the diminution of the satellite cell pool observed in muscular dystrophies (Bockhold et al., 1998; Cerletti et al., 2008). The mechanism controlling satellite cell self-renewal and the return to quiescence after proliferation remains incompletely understood. Satellite cell heterogeneity (Kuang et al., 2007), asymmetric inheritance of fate determinants (Conboy et al., 2007; Shinin et al., 2009) and modulation of signaling cascades such as Notch, Wnt and MAPK pathways (Jones et al., 2005; Kuang et al., 2007; Perez-Ruiz et al., 2008; Abou-Khalil et al., 2009) have all been proposed to control self-renewal. Precise control of growth factor sensitive-MAPK/RTK signaling is critical for myogenic fate decisions (Allen et al., 1984; Clegg et al., 1987; Fedorov et al., 1998; Jones et al., 2001). We propose that cycling muscle stem cells traverse through a growth factor-mediated checkpoint prior to returning to quiescence and in the absence of *Spry1* instead appropriate an apoptotic fate decision leading to a diminished pool of muscle stem cells.

Disruption of *Spry1* in all cells or specifically in adult Pax7^+^ satellite cells *in vivo* did not affect the formation or maintenance of the satellite cell pool under homeostatic conditions, however during muscle regeneration, the renewed Pax7^+^ satellite cell pool did not return back to homeostatic levels but instead was 50% smaller. In addition, targeting a heterogeneous population (Pax7-expressing and non-expressing) of myogenic progenitors *in vitro* led to a 50% reduction in cells that returned to quiescence *in vitro*. Together these data favor the hypothesis that *Spry1* is a major regulator of reversible quiescence and the ability of Pax7-derived satellite cells to self-renew. Furthermore, the data also strongly suggests that there is a level of heterogeneity within the renewing Pax7^+^ satellite cell compartment, whereby 50% of the Pax7^+^ satellite cell pool does not require *Spry1* for its renewal capability. Using a paradigm of repeated injuries to test self-renewal potential we demonstrate that the remaining (“Spry1-independent”) population of Pax7-derived satellite cells has self-renewal potential. Albeit this sub-population of Pax7^+^ cells is not sufficient or maybe not required to restore the pool of satellite cells back to levels observed prior to injury. Further experiments are required to determine the functional differences between the distinct “Spry1-dependent” and “Spry1-independent” populations.

A reciprocal balance between self-renewal and differentiation within a stem cell compartment is indicative of a single population of stem cells. Disruption of *Spry1* in Pax7^+^ cells affected the ability of the stem cell pool to renew, however we did not observe any reciprocal changes or alterations in the cells ability to differentiate either *in vitro* or *in vivo*. This suggests that *Spry1* function contributes to define the molecular heterogeneity in adult satellite cells. Muscle stem cell functional heterogeneity has been proposed based on satellite cells that progressed through a *Myf5* lineage during their developmental history (Kuang et al., 2007). Adult satellite cells that have not previously expressed *Myf5*, based on a readout from a *Myf5 Cre* reporter, had a greater self-renewal capacity, compared to satellite cells from a *Myf5* ancestry that had a greater capacity to differentiate. Disruption of *Spry1* within Pax7-derived satellite cells markedly affected their self-renewing potential, but not their differentiation; consistent with a requirement for *Spry1* in a sub-population of satellite cells that have high self-renewal potential but low differentiating potential. An alternative but not mutually exclusive hypothesis is that other *Spry* family members or other signaling pathways, such as the non-canonical Wnt cascade (Le Grand et al., 2009) coordinate to control self-renewal and differentiation, providing a level of heterogeneity and maybe cellular specificity at the level of the signaling pathway.

The co-ordination of cell fate decisions involving proliferation and subsequent differentiation of myogenic progenitors during muscle regeneration are controlled through an antagonistic relationship between Notch and canonical Wnt signaling (Brack et al., 2008). Based on re-expression of *Spry1* and the return to quiescence of Pax7^+^ satellite cell population in a coordinated manner during muscle regeneration raised the possibility that re-quiescence of the renewing stem cell pool was also coordinated in a stage-specific manner, occurring principally after the majority of satellite cell proliferation and early stages of differentiation has occurred. We speculate that *Spry* genes may fine-tune the dosage and/or duration of extrinsic signals within regenerating tissue to determine stem cell fate decisions for effective tissue homeostasis and repair.

In conclusion we provide direct genetic evidence that Sprouty1, a gene essential for embryonic cell fate determination, is an essential regulator of adult muscle stem quiescence and homeostasis of the self-renewing muscle stem cell pool during regeneration.

## Experimental Procedures Animals

Mice carrying the *Spry1* gene flanked by a pair of l*oxP* sites (*Spry1^flox^*) were used for tissue specific deletion studies (Basson et al., 2005). Mice with two copies of *Spry1^flox^* or with wild type *Spry1* (*Spry1^WT^*) were crossed with *Pax7-CreER^tm^;Spry1^flox/^*^+^ mice (Nishijo et al., 2009) to generate *Pax7-CreER^tm^;Spry1^flox/flox^* (Satellite cell- specific Spry1Null (SC-Null)) and Control littermates. Experimental animals were maintained on a CD1 background. Mice were bred to heterozygosity for *Pax7-CreER^tm^* and homozygosity with the *R26R-lacZ* reporter (Soriano, 1999). *Spry1^lacZ^* mice were maintained on a FVB background (Thum et al., 2008). Animals were housed and handled in accordance with the guidelines of the MGH subcommittee for animal research.

## Muscle Injury

Injury to whole TA/EDL muscle was made by injection of barium chloride (50 μl, 1.2%) into 30 sites in the lower limb. This produced an extensive injury resulting in homogenous damage and activation of satellite cells. A single intraperitoneal (IP) injections of BrdU (6 μg /gram mouse) was given followed by BrdU administered *ad libitum* in drinking water (2.5 mg/ml) for 7-12 days. Muscles were subjected to a secondary injury (50 μl, 1.2% of barium chloride) fifty days after an initial injury and allowed to recover for a further 50 days. For in vivo manipulation of ERK signaling U0126 (30 mg/kg diluted in 5% DMSO/PBS) or diluent alone (Schuh and Pahl, 2009) was administered via IP injection 11 and 12 days after initial muscle injury.

## Myogenic Cell Preparation

Single fiber cultures and satellite cell isolations were performed as described previously (Zammit et al., 2004; Brack et al., 2007). To generate ‘reserve cell’ cultures (Yoshida et al., 1998), low passage primary myoblasts were maintained in growth media (20% FBS, 5ng/ml bFGF in Ham's F-10 (Mediatech) and switched to differentiation media (3% HS in DMEM) for 3.5 days at high density (80-90% confluency). U0126 was added to cultures every 20 hours.

## Activation of Cre Recombinase

Low passage myoblasts were infected with adenovirus Ad5CMVCre-eGFP or Ad5CMV-eGFP-control (Gene Transfer Vector Core, University of Iowa) (diluted 1:1000 in growth media from a stock titer of 1×10^10^ pfu/ml) for 1.5 hours at 37°C. Cells were washed in PBS and incubated in fresh growth media for 18 hours and either fixed or re-plated in growth media at equal density for 24 hours and switched to fusion media for 1-4 days. Mice aged 3-4 months were given intraperitoneal (IP) injections of tamoxifen (TM) (300 μl, 10mg/ml, diluted in corn oil (Sigma)) daily for 3 days either prior to (12-14 days) or after injury (ranging from 7-9 to 18-20 days).

## Histology and Immunoflourescence

Muscles were dissected and embedded for cryostat sectioning as previously described (Brack et al., 2007). For analysis of β-gal activity, TA muscle was fixed in 2% PFA/0.2% gluteraldehyde and prepared as described previously (Brack et al., 2007). Tissue sections for immunohistochemistry were fixed in 4% PFA for 10 minutes, washed, permeabilized in 0.2% PBT and incubated in M.O.M. blocking reagent (10%, Vector) for 30 minutes then in 1% MOM, 10% GS/PBT, for 30 minutes. Sections were stained in Pax7 antibody overnight at 4°C. To reduce background staining of mouse antibody on regenerating mouse tissue, sections were washed, blocked and incubated in a ‘bridging antibody’ (rat anti-mouse IgG) at 1/500 for 3 hours at room temperature in addition to rabbit anti-MyoD, rabbit anti-Ki67 and chick anti-Laminin primary antibodies. Sections were washed, blocked and incubated in Alexa fluorophore conjugated species-specific anti-IgG antibodies with DAPI for 1 hr at room temperature. Tissue sections for BrdU detection were fixed in 4%PFA, washed in PBS and then antigen-retrieved with sodium citrate (10mM, 0.05% Tween in PBS) at 95°C for 30 minutes. Sections were washed in PBS for 30 minutes and blocked in 10% GS/PBT then incubated with anti-mouse Pax7 and anti-rat BrdU overnight at 4°C and secondary antibodies as described above. Immunofluorescence was performed on fixed cells and fibers (4% PFA, 10 min) after permeabilization (0.2% PBT; 10 min) and block (10% goat serum (GS) in PBT). Cells were incubated in primary antibodies overnight at 4°C. Cells were washed and blocked in 5% GS/PBT then incubated in Alexa fluorophore-conjugated antibodies and DAPI to visualize nuclei for 1 hr at room temperature. Antibodies and concentrations used are provided in Supplementary Material. Methods describing the quantification of muscle regeneration and is provided in Supplementary Material.

## Fluorescent Activated Cell Sorting (FACS)

To obtain highly purified myogenic cells, mononucleated cells were isolated from uninjured and regenerating muscle as described (Brack et al., 2009) with modifications. Cells were incubated in Sorting media (5% FBS, 10% BlokHen in Hams F10) for 10 min then incubated in anti-chick-Syn4 and anti mouse-integrin-α7 for 20 minutes. Cells were washed in sorting media and spun at 1500rpm for 5 min. Cells were stained in CD31-PE, CD45-PE, anti-chick Alexa688 and anti-mouse Alexa488 for 20 minutes. Myogenic cells had the following profile: Syn-4^+^, Integrin-α7^+^,CD31^-^,CD45^-^,PI^-^. Cells were sorted using FACS Aria (BD Biosciences). Protocol modified from (Sacco et al., 2008; Brack et al., 2009). Sorted cells for immunohistochemical analysis were cytospun immediately after collection. Description of Western blotting is provided in Supplementary Material.

## Real Time RT-PCR

Approximately 30,000 FACS sorted cells were collected from either uninjured or regenerating muscle and prepared for qRT-PCR analysis. First-strand cDNA was directly synthesized from each cell lysate by the SuperScript® III CellsDirect cDNA Synthesis Kit (Invitrogen). Quantitative real-time PCR was performed on the M×3000P qPCR system (Stratagene), with Brilliant SYBR Green qPCR master mix (Stratagene) using primers against Spry1 and GAPDH. Primers and thermal cycler conditions are detailed in the Supplementary Material. Analysis of genomic recombination of *Spry1* open reading frame was performed by PCR (Basson et al., 2005).

## Statistical Analysis

A minimum of 3 and up to 6 replicates was done for all experiments presented. Data are presented as means and standard errors of the mean. Comparisons between groups were done using a one way analysis of variance and a Tukey post hoc test. Comparisons within groups were done using a t-test with repeated measures. Differences were considered statistically significant at the *p* < 0.05 level.

## Supplementary Material

01
