# Supplementary material for: Sprouty1 Regulates Reversible Quiescence of a Self-Renewing Adult Muscle Stem Cell Pool during Regeneration
Source: Cell Stem Cell. Author manuscript; Available in PMC 2011 Feb 5. (PMC2846417; doi:10.1016/j.stem.2009.12.015)
Supplement: 01 [file NIHMS168182-supplement-01.pdf]

Inventory of Supplemental Information

Supplementary Experimental Procedures:

Contains information detailing experimental procedures excluded from the main body of manuscript.

Supplementary Figures:

Figure S1. Suporting data for figure 1 showing the expansion of myogenic cells and efficiency of muscle degeneration and regeneration after barium chloride injection.

Figure S2. Supporting data for Figure 2 showing FACS plots of purified myogenic cells and the Ct values of qRT-PCR from collected cells.

Figure S3 Supporting data for Figure 3 showing the high degree of genomic recombination after TM induced Cre excision.

Figure S4 Supporting data for Figure 4 showing that homozygous *Spry1*^{lacZ} allele functions as a null alle.

Figure S5 Supporting data for Figure 5 showing representative images of Myogenin+ cells in regenerating muscle.

Figure S6 Supporting data for Figure 6 showing Single muscle fiber suspension cultures and their complement of Pax7 and MyoD+ satellite cells.

Figure S7 Supporting data for Figure 7 showing Cre adenovirus-treated muscle cells and the high efficiency of recombination.

Figure S1

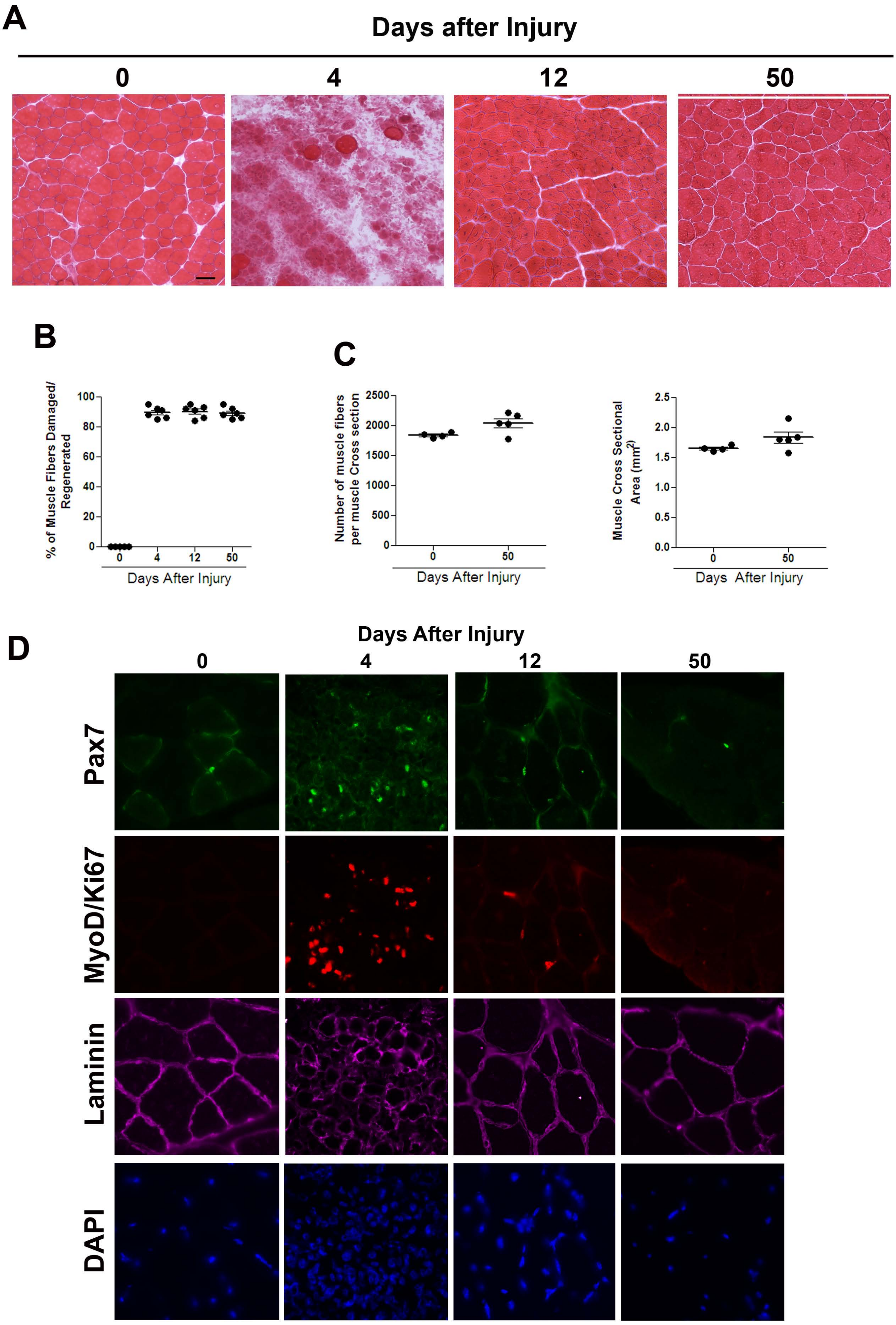

A
Days after Injury
0
4
12
50
B
% of Muscle Fibers Damaged/ Regenerated
100
80
60
40
20
0
0
4
12
50
Days After Injury
C
Number of muscle fibers per muscle Cross section
2500
2000
1500
1000
500
0
0
50
Days After Injury
Muscle Cross Sectional Area (mm^2)
2.5
2.0
1.5
1.0
0.5
0.0
0
50
Days After Injury
D
Days After Injury
0
4
12
50
Pax7
MyoD/Ki67
Laminin
DAPI

Figure S2

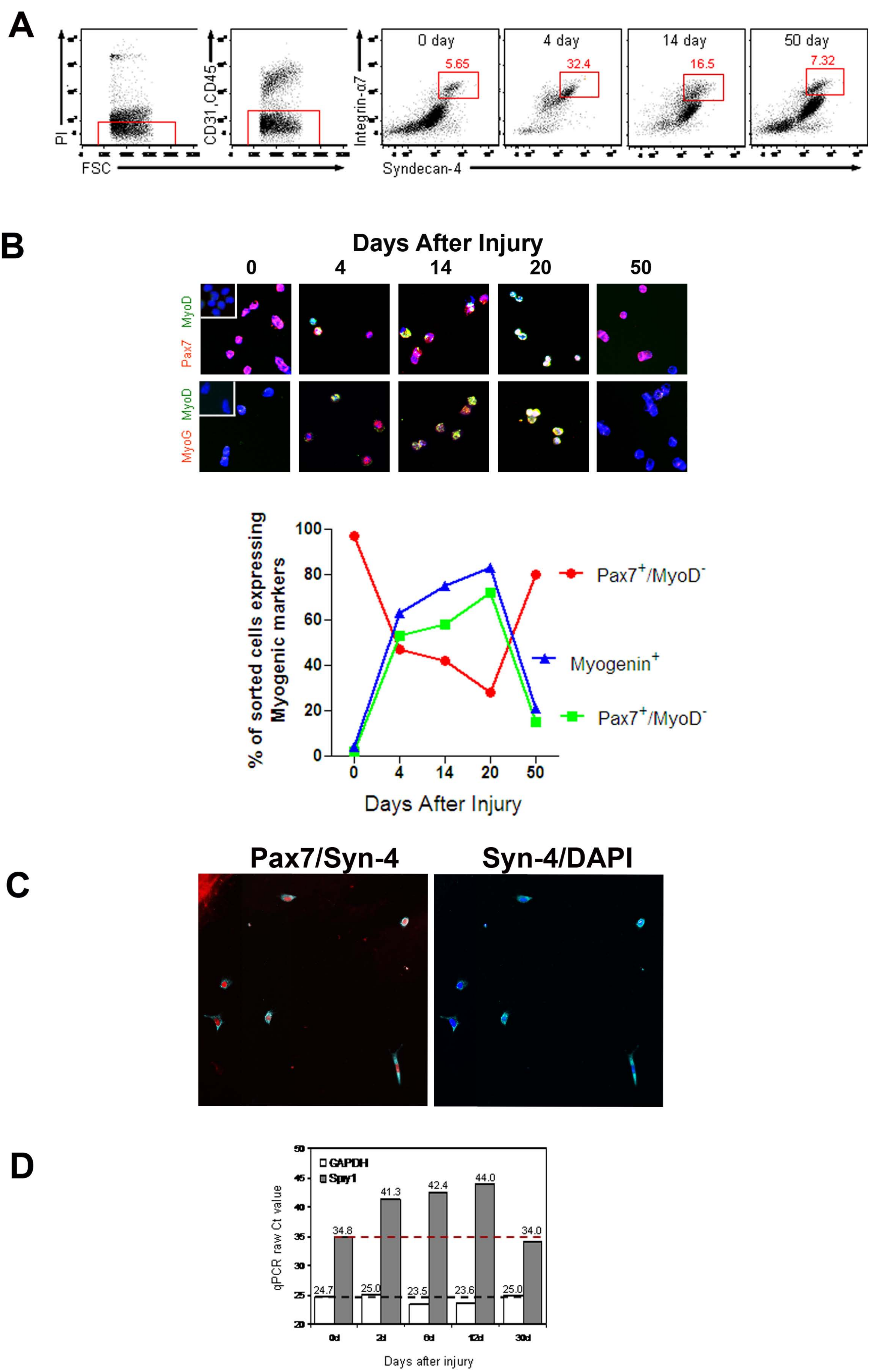
A
PI
FSC
CD31,CD45
Integrin-α7
Syndecan-4
0 day
5.65
4 day
32.4
14 day
16.5
50 day
7.32
B
Days After Injury
0
4
14
20
50
Pax7 MyoD
MyoG MyoD
% of sorted cells expressing Myogenic markers
Days After Injury
Pax7+/MyoD-
Myogenin+
Pax7+/MyoD-
C
Pax7/Syn-4
Syn-4/DAPI
D
GAPDH
Spry1
qPCR raw Ct value
34.8
41.3
42.4
44.0
34.0
24.7
25.0
23.5
23.6
25.0
0d
2d
6d
12d
30d
Days after injury

Figure S3.

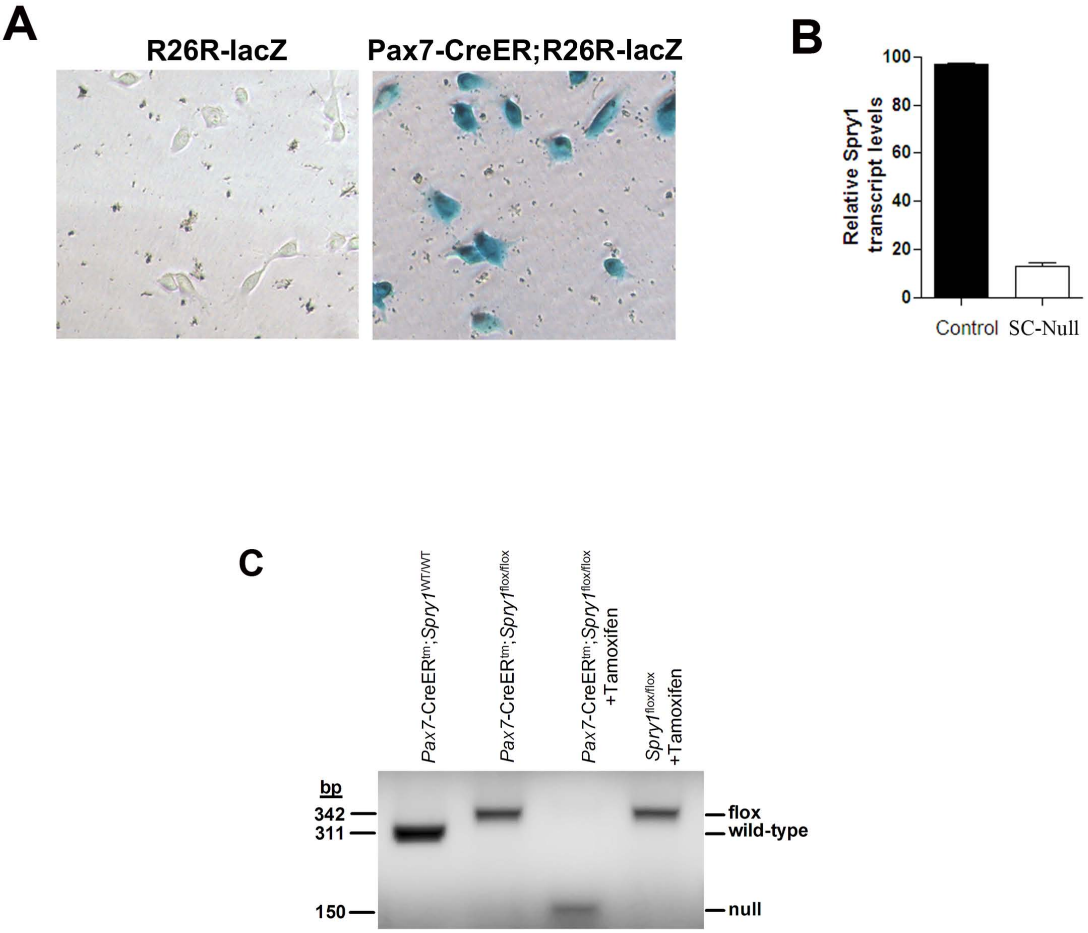
A
R26R-lacZ
Pax7-CreER;R26R-lacZ
B
Relative Spry1 transcript levels
100
80
60
40
20
0
Control
SC-Null
C
Pax7-CreERtm;Spry1WT/WT
Pax7-CreERtm;Spry1flox/flox
Pax7-CreERtm;Spry1flox/flox +Tamoxifen
Spry1flox/flox +Tamoxifen
bp
342
311
150
flox
wild-type
null

Figure S4.

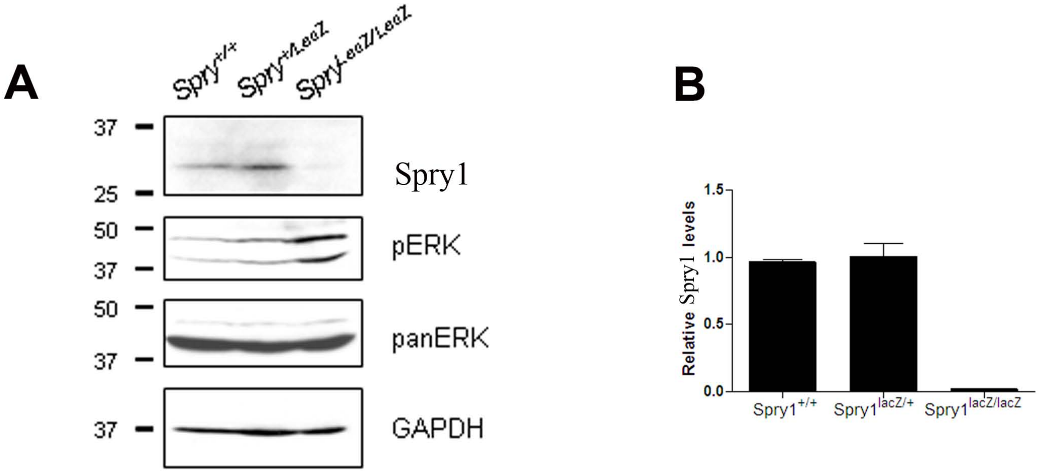
A
Spry+/+
Spry+/LacZ
SpryLacZ/LacZ
37
25
Spry1
50
37
pERK
50
37
panERK
37
GAPDH
B
Relative Spry1 levels
1.5
1.0
0.5
0.0
Spry1+/+
Spry1lacZ/+
Spry1lacZ/lacZ

Figure S5.

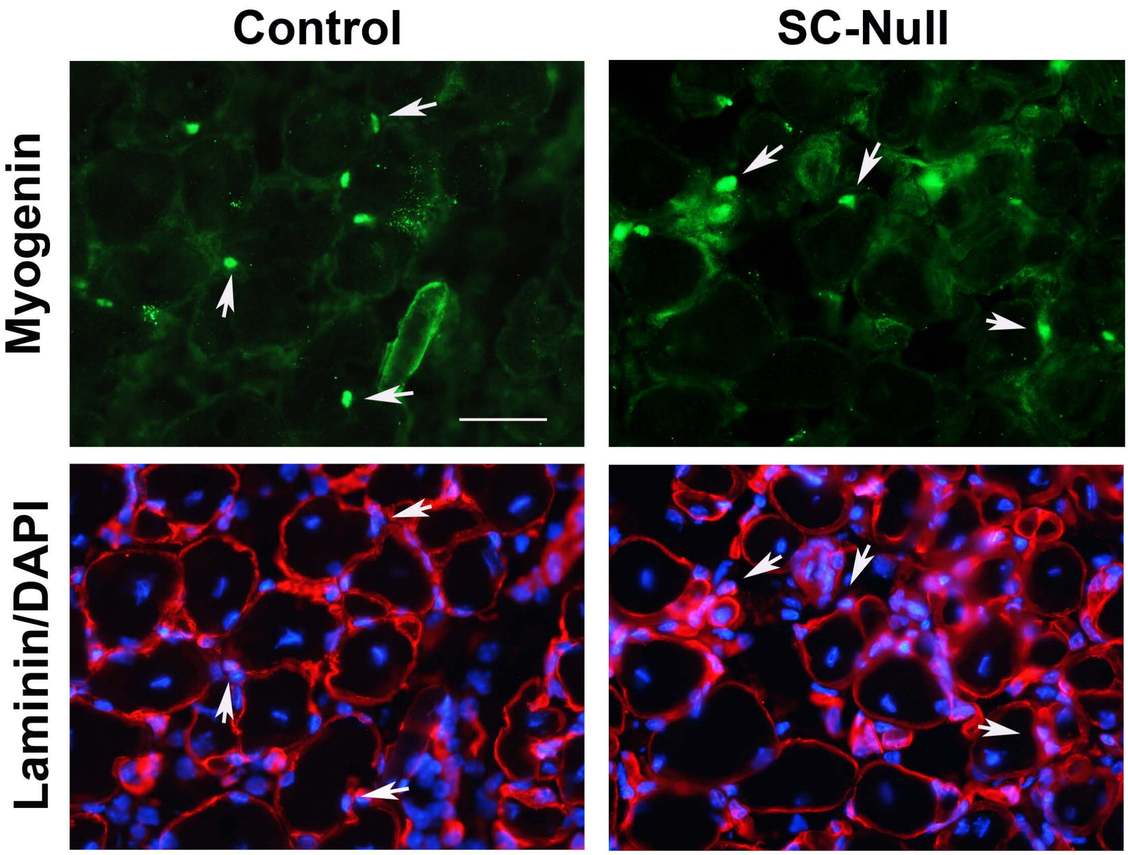

Figure S6.

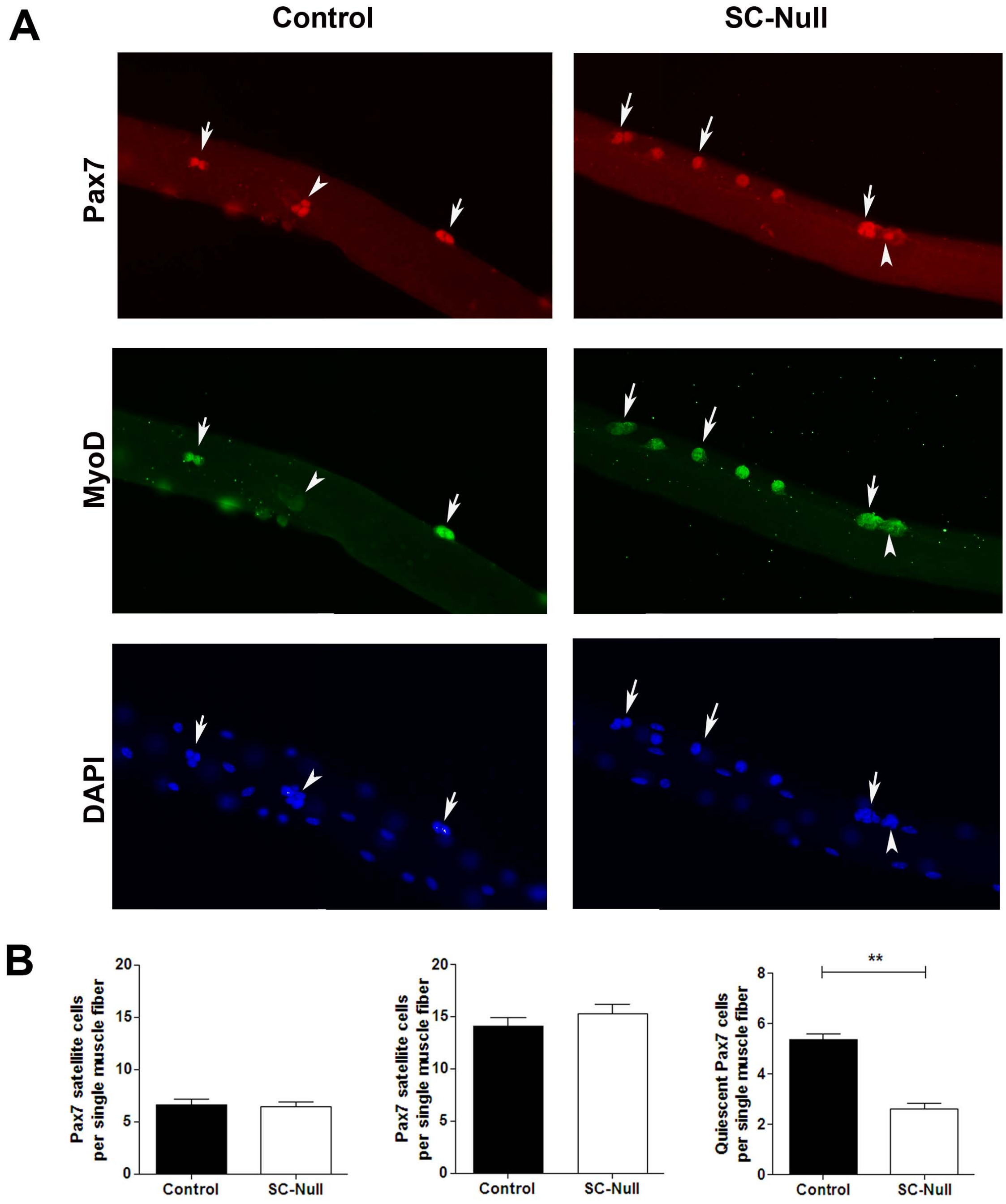
A
Control
SC-Null
Pax7
MyoD
DAPI
B
Pax7 satellite cells per single muscle fiber
20
15
10
5
0
Control
SC-Null
Pax7 satellite cells per single muscle fiber
20
15
10
5
0
Control
SC-Null
Quiescent Pax7 cells per single muscle fiber
8
6
4
2
0
**
Control
SC-Null

Figure S7

A

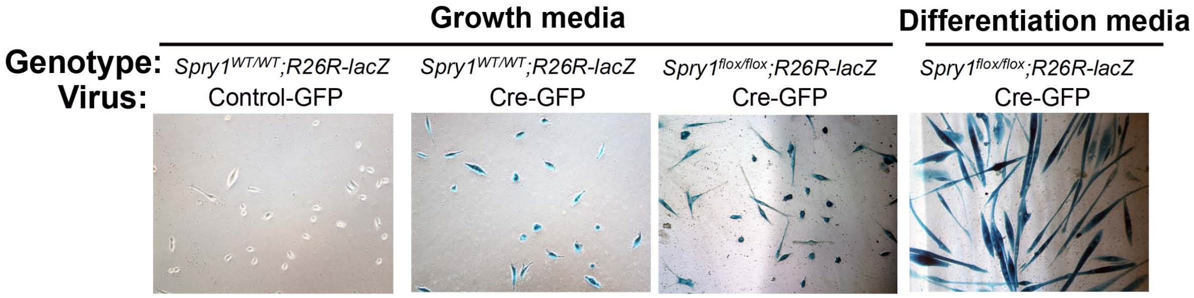
Growth media
Differentiation media
Genotype: Spry1WT/WT;R26R-lacZ Spry1WT/WT;R26R-lacZ Spry1flox/flox;R26R-lacZ Spry1flox/flox;R26R-lacZ
Virus: Control-GFP Cre-GFP Cre-GFP Cre-GFP

B

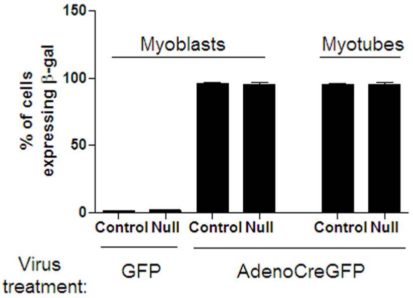
150
100
50
0
% of cells expressing β-gal
Myoblasts
Myotubes
Control Null Control Null Control Null
Virus treatment:
GFP
AdenoCreGFP

SOM Figure legends.

Figure S1. A method to achieve reproducible muscle injury. (A) Muscle was injured with 50 µl of barium chloride (1.2%) with 30 multiple needle punctures. Transverse sections were collected from uninjured and regenerating muscle and stained with Haematoxylin and Eosin. (B) The percentage of muscle fibers damaged using this injury protocol. Muscles that had less than 80% of all fibers damaged were excluded from further analysis. (C) The total number of muscle fibers (left panel) in the uninjured and regenerated (50 days after injury) muscle and the muscle cross sectional area size in the midbelly of the uninjured and regenerated TA muscle (right panel). Scale bar; 100 µm. (D) The pool of quiescent Pax7 satellite cells returns to homeostasis during adult muscle regeneration. Sections of regenerating and uninjured TA muscles were stained against Pax7 (green), MyoD and Ki67 (red) and laminin (magenta). DAPI stains nuclei blue.

Figure S2. Identification of highly-enriched myogenic cells isolated from uninjured and regenerating muscle. (A) Representative FACS plots from cells isolated from TA/EDL muscles that were injured and left to regenerate for 4, 14 and 50 days or remained uninjured (0 day) and stained with propidium iodide (PI) anti-Syndecan-4-APC (Syn4), anti-Integrin-α7-FITC (Int7), anti-CD31-TRITC, and anti-CD45-TRITC antibodies. Cells gated on $Syn4^{+}/Int7^{+}/CD31^{-}/CD45^{-}/PI^{-}$ were collected and used for further study of myogenic cells. Red box within each plot indicates the collected fraction and the percentage of cells collected from the $CD31^{-}/CD45^{-}/PI^{-}$ fraction during regeneration. (B) A fraction of sorted cells (n=2000) from the positive selected gate (in panel A) were cytospun and stained for Pax7/MyoD and MyoD/Myogenin and DAPI

immediately after sorting. Inset shows cells collected from the negative selected gate ($Syn4^-/Int7^-/CD31^-/CD45^-/PI^-$) were negative for myogenic markers. Histogram shows that 99% of sorted cells express markers of myogenicity within different stages of myogenesis: Quiescent ($Pax7^+/MyoD^-$) proliferating ($Pax7^+/MyoD^+$) and differentiating ($MyoD^+/Myogenin^+$). (C) Sorted myogenic cells were cultured in growth media for 24 hours, fixed and stained for Pax7 (red) and Syn-4 (cyan) and DAPI. (D) Transcript abundance was expressed as Ct values for Spry1 and GAPDH from sorted cells isolated from uninjured and regenerating muscle shown in Figure 3A. Dashed line indicates the baseline that time-points were normalized against.

Figure S3. Efficiency of *Pax7-CreER^{tm}*-mediated recombination in muscle satellite cells. (A) *R26R-lacZ* and *Pax7CreER^{tm};R26R-lacZ* mice were treated with TM and left to recover for 14 days. Sorted myogenic cells were obtained as described in S2 and plated in culture for 24 hours, fixed and stained with X-gal. (B) *Spry1* transcript abundance was determined from sorted myogenic cells from TM-treated *Spry1$^{flox/flox}$;R2626-lacZ* (Control) and *Pax7CreER^{tm};Spry1$^{flox/flox}$;R26R-lacZ* (SC-Null) mice. *Spry1* transcript level in cells was normalized to GAPDH levels and expressed relative to Control cells. Average Ct values: GAPDH: 30.32 and 30.49; Spry1: 41.25 and 43.81, from Control and SC-Null, respectively. (C) Sorted myogenic cells (20,000) were collected from corn oil and TM-treated Control and SC-Null mice and genomic DNA was tested for recombination of the *Spry1* locus.

Figure S4. Loss of Spry1 protein using a Sprouty1 knockin allele.

(A) Western blot analysis of Spry1 and ERK expression from low passage (P2) myogenic progenitors from *Spry1*$^{+/+}$ (Control), *Spry1*$^{+/lacZ}$ and *Spry1*$^{lacZ/lacZ}$ mice. (B) Quantitation of Spry1 protein levels relative to GAPDH. Data expressed as mean ± sem.

Figure S5. The number of differentiated myogenic cells during regeneration.
Representative image of regenerating muscle sections. Muscles were injured and left to regenerate for 12 days, fixed and stained with antibodies against Myogenin and Laminin. DAPI stains nuclei blue. Scale bar; 40 μm.

Figure S6. Self-renewal of Pax7 cells from single muscle fiber cultures.
(A) Single muscle fiber cultures were generated from TM-treated SC-Null (*Pax7creER*^{tm};*Spry1*$^{flox/flox}$) and Control (*Spry1*$^{flox/flox}$) mice and left in plating media for either 42 or 72 hours and fixed. Cultures were stained with anti-Pax7, MyoD, Ki67 and DAPI. (B) The total number of Pax7 cells at 42 hours (left) and 72 hours (middle) and the percentage of quiescent Pax7 cells at 72 hours (right). A minimum of 30 fibers were counted within each experiment and expressed as mean ± sem. Experiments done in triplicate.

Figure S7. Cre adenovirus induced recombination in myogenic progenitors in vitro.
(A) Primary myoblast cultures were generated from *Spry1*$^{WT/WT}$;*R26R-lacZ* and *Spry1*$^{fox/flox}$;*R26R-lacZ* mice and treated with Control GFP adenovirus or Cre adenovirus for 1.5 hours, left to recover in growth media and either fixed or switched to differentiation media for 3 days to generate myotubes and then fixed. Cells were stained

with X-gal to estimate Cre-induced recombination. (B) The percentage of X-gal$^+$ myoblasts or differentiated myotubes. A minimum of 500 cells were counted within each experiment and expressed as mean ± sem. Experiments done in triplicate.

Supplemental Experimental Procedures

Reagents and Antibodies

The source and concentration of antibodies: β-gal antibody (1/3000, Cappel), Rat anti-BrdU (1/500, Abcam), rabbit anti-ki67 (1/200, Abcam) mouse anti-Pax7 (1/100, DSHB), rabbit anti-MyoD (1/100, Santa Cruz), mouse anti-MyoD (1/200, BD), mouse anti-Myogenin (1/200, BD), Cleaved Caspase-3 (1/500, Cell Signaling Technologies), rat anti-BrdU (1/500, BD), mouse anti-BrdU (1/500), chick anti-Laminin (1/5000, Abcam) chick anti-Syn-4 (1/200 for FACS, 1/2000 for immunohistochemistry, kindly provided by Dr. Brad Olwin (University of Calorado), mouse anti-Integrin-α7,(1/200, MBL), CD31-PE and CD45-PE (1/200, BD). The corresponding species-specific Alexa-conjugated (488, 546, 647) secondary antibodies (Molecular probes) were used at 1/2000 for immunohistochemistry or 1/200 for FACS. TUNEL (Roche *In situ* Death Detection Kit, Fluorescein). Rabbit anti-Sprouty 1 (1:500, Invitrogen), rabbit anti-GAPDH (1:8000, Applied Biosystems) panERK and phosphorylated ERK (1/1000, BD-both at 1/1000) were used for Western blot analysis. Tamoxifen was from Sigma (St. Louis, MO). U0126 was used as a MEK inhibitor for in vitro studies (3-10 μM diluted in DMSO, Calbiochem). Basic FGF2 was from R&D Systems (Minneapolis, MN).

Determining the extent of muscle injury

Muscle was injured and the total number of non-injured fibers (non-centrally nucleated) at earlier stages of regeneration (4 days after injury) was compared to the non-injured contra-lateral muscle. After 12-50 days of regeneration the ratio of regenerating (centrally-nucleated) and non-regenerating were quantified and normalized to uninjured

contra-lateral control. Muscles were only included in subsequent analysis if the percentage of regenerating fibers was between 85-95%.

Determination of Muscle size, Fiber size and Fiber number

The cross-sectional area of serial muscle sections and the number and size of regenerating muscle fibers (denoted by $DAPI^+$ central nucleation) and non-regenerating fibers from uninjured muscle were quantified. Muscle cross section was quantified through a x2 objective at three regions in the mid-belly of each muscle. Images were collected and total muscle fiber number was quantified. Average muscle fiber size was determined based on Laminin staining using Nikon Elements software. A minimum of 1000 fibers were quantified per muscle. A minimum of 10 sections per muscle was analyzed.

Analysis of Satellite cells and their progeny

Muscles sections were stained with a cocktail of antibodies to determine the number of $Pax7^+$ satellite cells that were quiescent ($Pax7^+$, $ki67^-$,$MyoD^-$) or cycling ($Pax7^+$, $ki67^+$,$MyoD^+$) underneath the basal lamina ($laminin^+$) at different times after muscle injury. The number of satellite cells and their progeny was determined based on the number of $Pax7^+$ cells per 10 µm muscle serial section. To minimize potential variation in satellite cell numbers based on muscle size, satellite cell quantification was only conducted from muscle sections of equal size. The total number of $Pax7^+$ cells was quantified in a minimum of 10 serial sections per muscle in 3 separate regions from the mid-belly of the muscle. A minimum of 500 $Pax7^+$ cells were counted per muscle. The

number of Pax7 cells was quantified on freshly isolated single EDL muscle fibers. A minimum of 20 muscle fibers were counted per animal. A minimum of 100 $Pax7^{+}$ cells were counted per animal. Reserve cell cultures were stained with a panel of antibodies to characterize myogenic cells in quiescence ($Pax7^{+}$, $MyoD^{-}$/$ki67^{-}$), apoptosis (Caspase-3^{+}/$MyoD^{+}$) and differentiation ($Myogenin^{+}$). Cells were scored until a minimum of 500 cells were counted. All experiments were done in triplicate.

Western blot

Muscle cells were homogenized in lysis buffer (25 mM Tris-HCl, pH 6.8, 3.5% SDS, 5% β-mercaptoethanol, protease inhibitor cocktail, 1:100, phosphatase inhibitor cocktails 1 and 2, 1:100, Sigma) and mixed with sample. Twenty microgram total protein of each sample was subjected to SDS-PAGE and western blotting, following standard protocols. Primary antibodies were diluted in TTBS (20 mM Tris, pH 7.5, 0.1 M NaCl, 0.1% Tween 20) containing 5% dry milk, and incubated with the blot overnight at 4°C. After incubation with HRP-conjugated secondary antibody (1:10,000, Pierce), immunoreactivity was detected with ECL system (GE) according to manufacturer instruction. Chemiluminescent signals was be obtained after exposure to a BioMax film (Kodak) and analyzed with image processing program ImageJ (National Institutes of Health).

RT-PCR

Primers for Spry1: GAGGCCGAGGATTTCAGATGCA and CTGAATCACCACTAGCGAAGTGT and GAPDH: CACTGAGCATCTCCCTCACA

and GTGGGTGCAGCGAACTTTAT. All reactions for Real time qPCR were performed using the following thermal cycler conditions: 95°C for 10 min followed by 45 cycles of a 3 step reaction; denaturation at 95°C for 30 sec, annealing at 55°C for 30 sec and extension and data collection at 72°C for 30 sec. Sprouty1 expression was normalized to GAPDH. Absolute Ct values are displayed in Supplementary figures.
